# Luteapyrone, a Novel ƴ-Pyrone Isolated from the Filamentous Fungus *Metapochonia lutea*

**DOI:** 10.3390/molecules26216589

**Published:** 2021-10-30

**Authors:** Roman Labuda, Markus Bacher, Hannes Gratzl, Maria Doppler, Alexandra Parich, Mohammed Aufy, Rosa Lemmens-Gruber, Rainer Schuhmacher, Kathrin Rychli, Martin Wagner, Thomas Rosenau, Joseph Strauss, Christoph Schüller

**Affiliations:** 1 Unit of Food Microbiology, Department for Farm Animals and Veterinary Public Health, Institute of Food Safety, Food Technology and Veterinary Public Health, University of Veterinary Medicine Vienna, Veterinaerplatz 1, 1210 Vienna, Austria; Kathrin.rychli@vetmeduni.ac.at (K.R.); martin.wagner@vetmeduni.ac.at (M.W.); 2 Core Facility Bioactive Molecules, Screening and Analysis and Research Platform Bioactive Microbial Metabolites (BiMM), Konrad Lorenz Strasse 24, 3430 Tulln an der Donau, Austria; markus.bacher@boku.ac.at (M.B.); hannes.gratzl@boku.ac.at (H.G.); maria.doppler@boku.ac.at (M.D.); rainer.schuhmacher@boku.ac.at (R.S.); joseph.strauss@boku.ac.at (J.S.); christoph.schueller@boku.ac.at (C.S.); 3 Department of Chemistry, University of Natural Resources and Life Sciences, Vienna (BOKU), Konrad Lorenz Strasse 24, 3430 Tulln an der Donau, Austria; thomas.rosenau@boku.ac.at; 4 Department of Agrobiotechnology (IFA-Tulln), Institute of Bioanalytics and Agro-Metabolomics, University of Natural Resources and Life Sciences, Vienna (BOKU), Konrad Lorenz Strasse 20, 3430 Tulln an der Donau, Austria; alexandra.parich@boku.ac.at; 5 Department for Pharmacology and Toxicology, University of Vienna, Althanstrasse 14, 1090 Vienna, Austria; mohammed.aufy@univie.ac.at (M.A.); rosa.lemmens@univie.ac.at (R.L.-G.); 6 Johan Gadolin Process Chemistry Centre, Åbo Akademi University, Porthansgatan 3, FI-20500 Turku, Finland; 7 Department of Applied Genetics and Cell Biology, Institute of Microbial Genetics, University of Natural Resources and Life Sciences, Vienna (BOKU), Konrad Lorenz Strasse 24, 3430 Tulln an der Donau, Austria

**Keywords:** actinopyrones, fungal metabolites, verticipyrone, *Verticillium*-like species

## Abstract

In the process of screening for new bioactive microbial metabolites we found a novel ƴ-pyrone derivative for which we propose the trivial name luteapyrone, in a recently described microscopic filamentous fungus, *Metapochonia lutea* BiMM-F96/DF4. The compound was isolated from the culture extract of the fungus grown on modified yeast extract sucrose medium by means of flash chromatography followed by preparative HPLC. The chemical structure was elucidated by NMR and LC-MS. The new compound was found to be non-cytotoxic against three mammalian cell lines (HEK 263, KB-3.1 and Caco-2). Similarly, no antimicrobial activity was observed in tested microorganisms (gram positive and negative bacteria, yeast and fungi).

## 1. Introduction

The microscopic fungus, *Metapochonia lutea* (Ascomycota, Hypocreales, Clavicipitaceae), has been isolated and described as a novel taxon during a mycological survey of environmental samples from a coastal-zone water of the Danube river in Tulln an der Donau, Austria (EU) [1]. In general, the genus *Metapochonia* (and related *Pochonia*) comprises species living mainly in soil, often with a potential to parasitize nematode cysts. Their ecology, distribution and potential application as biological control agents against nematodes has been comprehensively reviewed [2,3,4]. As indicated earlier [5], many novel bioactive compounds can be expected to be discovered from these species. Indeed, these fungi are producing secondary bioactive metabolites with antifungal, antiviral and anthelmintic activities [6,7].

In our search for novel bioactive metabolites produced by *M. lutea* BiMM-F96/DF4 (ex-type culture) several potentially active compounds (e.g., succinic acid, phenyllactic acid, vanillic acid, anthraquinone-related compounds) were identified (data not shown) including a compound belonging to the actinopyrone γ-pyrone family [8]. These γ-pyrone compounds, actinopyrones A, B and C were isolated for the first time in 1986 from an actinomycete *Streptomyces pactum*, followed a few years later by the discovery of kalkipyrone, which was recovered from the marine cyanobacteria *Lyngbya majuscule* and *Tolypothrix* sp. [8]. A first fungal metabolite of this γ-pyrone family, verticipyrone has been isolated from *Verticillium* sp. FKI-1082 (Ascomycota, Hypocreales, Clavicipitaceae) [9]. The recent reports on description of new structurally related γ-pyrones, namely fusapyrones and fusaresters A–E are connected to fungal genus *Fusarium* (Ascomycota, Hypocreales, Nectriaceae) species [10,11,12] and acrepyrone A to endophytic fungus *Acremonium citrinum* SS-g13 [13]. 

Here we report a new ƴ-pyrone denominated luteapyrone. We describe the fungal cultivation, the subsequent compound isolation, bioactivity tests, and structure elucidation. 

## 2. Results

**Figure 1:**
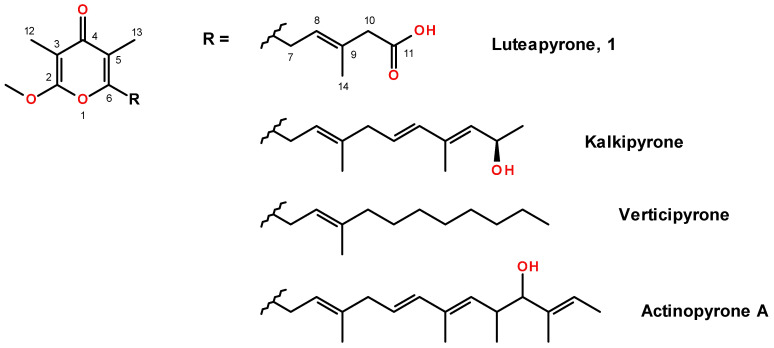
Chemical structures of luteapyrone (**1**) and related ƴ-pyrones [9].

**Figure 2:**
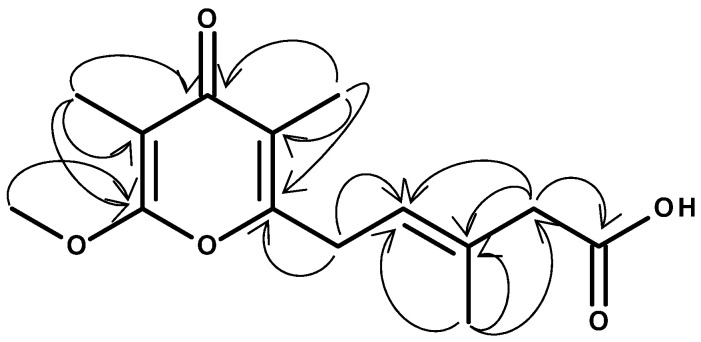
Detected HMBC crosspeaks of luteapyrone (**1**).

**Figure 3:**
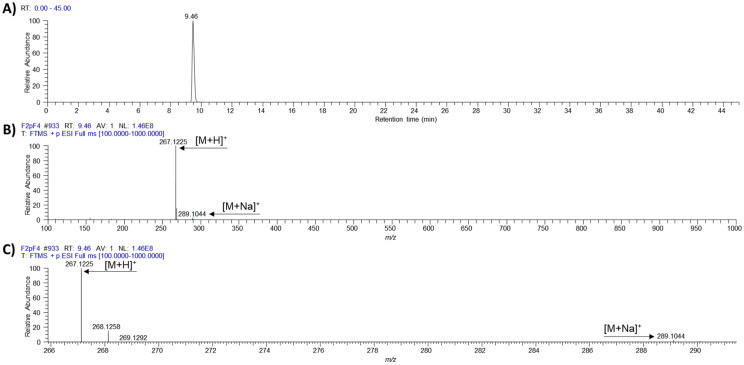
(**A**) Extracted ion chromatogram of [M + H]^+^ (*m/z* 267.1227 ± 5 ppm); (**B**,**C**) HR-FullScan mass spectrum at RT 9.46 min for full detected mass range (*m/z* 100–1000 (**B**)) and zoomed mass range (*m/z* 266–291 (**C**)) of luteapyrone (**1**).

**Figure 4:**
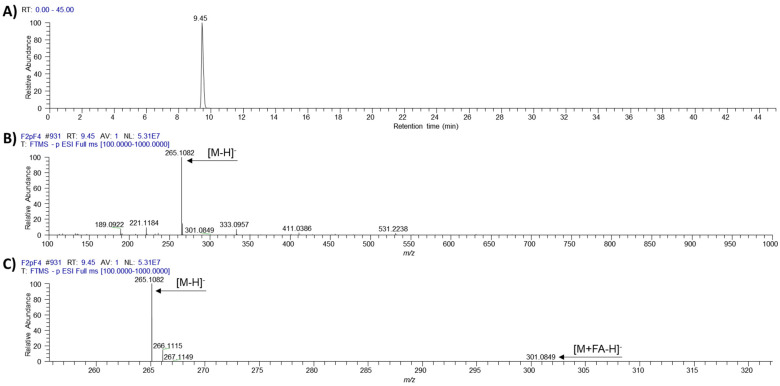
(**A**) Extracted ion chromatogram of [M − H]^−^ (*m/z* 265.1081 ± 5 ppm); (**B**,**C**) HR-FullScan mass spectrum at RT 9.45 min for full detected mass range (*m/z* 100–1000 (**B**)) and zoomed mass range (*m/z* 256–322 (**C**)) of luteapyrone (**1**).

**Figure 5:**
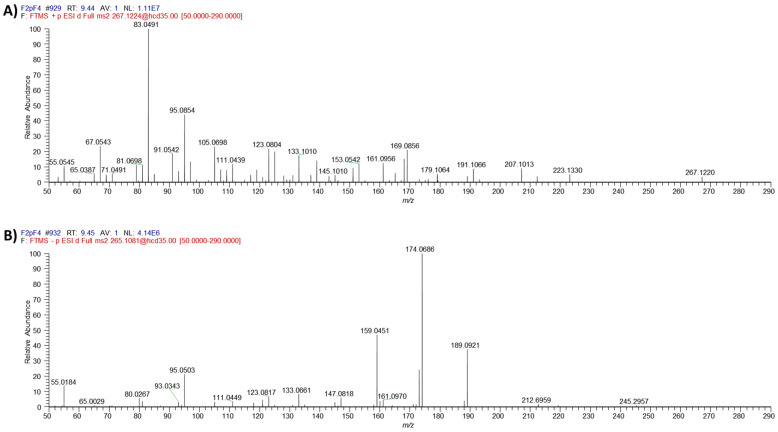
MS/MS fragmentation spectra of [M + H]^+^ (**A**) and [M − H]^−^ (**B**) of luteapyrone (**1**).

The novel ƴ-pyrone, luteapyrone (**1**) was obtained after purification as yellow-brownish oil. Its molecular formula was determined as C_14_H_18_O_5_ based on its [M + H]^+^ peak at *m/z* = 267.1229 (calcd 267.1227 for C_14_H_19_O_5_). The ^1^H NMR spectra showed the resonances of one methoxyl group at δ_H_ 4.02 ppm and three aliphatic methyl groups at δ_H_ 1.81, 1.86, and 1.95 ppm (Table 1). In addition, a singlet methylene group at δ_H_ 3.05 ppm and an olefinic proton at δ_H_ 5.44 ppm coupled to an additional methylene group at δ_H_ 3.48 ppm were detected. These findings were complemented by ^13^C data in combination with 2D NMR spectra. The ^13^C spectrum is characterized by 14 signals: besides the presence of the methoxyl group, three aliphatic methyls and two methylene groups, also six olefinic carbons (five quarternary and, in accordance with the ^1^H NMR spectrum, one CH), one carboxylic resonance at δ_C_ 175.7 ppm and one low-field resonance at δ_C_ 183.2 ppm were detected. The individual structural elements were unequivocally connected by different HMBC crosspeaks. The methoxyl group showed a crosspeak to the quarternary carbon at δ_C_ 164.7 ppm, a chemical shift attributable to a masked ester moiety. This carbon resonance gave also a long-range crosspeak to the methyl group at δ_H_ 1.81 ppm. This resonance in turn, in addition to a second methyl group at δ_H_ 1.96 ppm, could be connected to the low-field carbon at δ_C_ 183.2 ppm. This shift indicated the presence of cross-conjugated, strongly deshielded carbonyl group, typical for e.g., pyrones, chalcones or quinones. Analyses of all the remaining HMBC cross-peaks finally deduced the sidechain of the pyrone leading to structure **1** for the isolated metabolite (Figure 1). ^1^H and ^13^C chemical shifts are listed in Table 1, whereas relevant HMBC cross-peaks are shown in Figure 2. LC-HRMS analysis revealed distinct signals at a retention time of 9.45 min. Extracted ion chromatograms of [M + H]^+^ and [M − H]^−^ and the respective full scan mass spectra in positive and negative ionization mode as well as the MS/MS fragmentation pattern of compound **1** are depicted in Figure 3, Figure 4 and Figure 5. 

The new compound luteapyrone was evaluated for its cytotoxic activity in vitro against two cancer cell lines (KB-3.1 and Caco-2) as well as one non-cancer cell line (HEK-293). These tests revealed no significant cytotoxic effects in a concentration range of 1.3 to 85.1 µg/mL (5–320 µM), and IC_50_ was not reached even at the highest test concentration of 320 µM (Table S1, Supporting Information). Furthermore, its antimicrobial activity was evaluated against the Gram-positive bacterium *Staphylococcus aureus* ATCC 6538, the Gram-negative bacteria *Escherichia coli* ATCC 25922, *Klebsiella pneumoniae* ATCC 10031 and *Pseudomonas aeruginosa* ATCC 9027, the yeast *Candida albicans* ATCC 10231, and the filamentous fungi *Aspergillus fumigatus* RL 578), *Fusarium oxysporum* (RL 108) and *Fusarium solani* (RL 585). However, no antimicrobial activity was found against any of the tested microorganisms even at the highest concentration of 262.0 µg/mL (985 µM) (Table S2, Supporting Information). 

## 3. Discussion

The ƴ-pyrone-based natural products constitute a large class of biologically active compounds are found in all areas of life. They can be classified into three major ƴ-pyrone natural product families: the colletotrichins, the nitrophenyl ƴ-pyrones and the actinopyrones [8]. Compound **1** is structurally related to this last group, the actinopyrones, described by Yano et al. [9], or also to verticipyrone and kalkipyrone [9] (Figure 1). Whereas the sidechains of the actinopyrone and verticipyrone are composed of 11–13 carbons, luteapyrone contains a much shorter sidechain of only five carbons. In addition, the terminus is oxidized to a carboxylic acid, which seems to be an uncommon structural motif for ƴ-pyrones. So far, all structurally similar members of this ƴ-pyrone group (actinopyrones) have been isolated from bacteria [8,14], with two exceptions: verticipyrone has been isolated from the filamentous fungus *Verticillium* sp. [10] and recently described acrepyrone A from *Acremonium citrinum* [13]. In fact, the fungal genus *Verticillium* is closely related to *Pochonia* and *Metapochonia*, and only recently a taxonomic consensus concerning these related genera was achieved [15]. According to a thorough study on chemotaxonomy of *Pochonia* and other *Verticillium*-like anamorphs [6], ƴ-pyrone has not been produced by any of the 48 strains within 19 *Verticillium*-like species. Therefore, we suppose that production of luteapyrone by *M. lutea* might be species-specific, and not widely distributed even within the genus *Metapochonia* and/or related *Verticillium*-like anamorphic fungi. 

The physiologically active substances actinopyrone A, B and C have coronary vasodilating and antimicrobial activities as e.g., they inhibit the growth of *Helicobacter pylori* at a MIC value of 0.1 ng/mL. Toxicity to brine shrimps and goldfish was reported for kalkipyrone [8]. Verticipyrone was found to act anthelmintically by inhibiting NADH-fumarate reductase (NFRD) of *Ascaris suum* (roundworm) with an IC_50_ value of 0.88 nM [9]. Furthermore, it showed anthelmintic activity against *Caenorhabditis elegans* and *Artemia salina*, suggesting its use as an antiparasitic agent [8]. 

Unfortunately, the new compound luteapyrone was not effective in any of the tests performed in our study, showing neither antimicrobial nor cytotoxic effects. Similar results were reported for a structurally related acrepyrone A showing no antimicrobial or cytotoxic effects in the tests performed [13]. However, due to its structural similarity with other ƴ-pyrones within the group of actinopyrones, an insecticidal or antiparasitic activity [8,9] can be expected. Antiobesity activities of the actinopyrones have been reported [16,17].

## 4. Methods

### 4.1. Fungal Isolation and Taxonomy

The microscopic filamentous fungus *Metapochonia lutea* BiMM-F96/DF4 was found in a sample of water from the Danube river in Tulln (Austria) collected in July 2017. Detailed information about origin, isolation and taxon has been reported earlier [1]. hylogenetically, the combination of the internal transcribed spacer region (ITS) and translation elongation factor-1 α gene (tef-1 α) sequences resulted in resolving *M. lutea* in the monophyletic *Metapochonia* clade, with *M. rubescens* as the closest relative species. Molecular markers (DNA sequences) of the fungus are deposited in GenBank for MF983717 (ITS), MF983718 (tef-1α), MG182375 (tubB).

### 4.2. Fermentation and Extraction

The fungal spore suspension (5.0 × 10^6^ spores/mL) was obtained after 7 days cultivation of the fungus (*M. lutea* BiMM-F96/DF4) on a potato dextrose agar (PDA, Van Waters and Rogers (VWR) International, Leuven, Belgium, Austria). Five colony plugs were cut (each ca 1 × 1 cm) and thoroughly mixed (on vortex for 2 min) with 30 mL of physiological solution (0.9 % NaCl) in a sterile, 50 mL Falcon tube. In total, 2 L of yeast extract sucrose agar medium (YES, Samson et al., 2000) spread over appr. 80 Petri plates, was used for production of secondary metabolites. The production medium (YES) was modified by reducing the total content of sucrose (from 15% to 5%) and agar-agar (from 2% to 0.5%). Each plate was inoculated with 100 µL of spore suspension in three parallel streaks at the central and sub-central part of the plate. The plates were cultivated in perforated plastic bags for 14 days at 25 °C in the dark. At the end of the cultivation, the plates were checked for purity, cut into small pieces and the whole content of the plates (fungal colonies with medium) was harvested into a 5 L glass flask. The material was then mixed with 2 L of ethyl acetate. After vigorous stirring for 2 min in three subsequent steps (with ca 20 min in between), the mixture was filtered through a steel sieve in order to separate the solid particles (fungus and medium). The remaining residual water (generated by condensation of the water on plates during fungal growth) was removed by the addition of 10 g of anhydrous sodium sulphate. The organic phase was then filtered through a filter paper (270 mm i.d., Macherey-Nagel, Düren, Germany) and concentrated under reduced pressure at 45 °C (Büchi Rotavapor R-114, Flawil, Switzerland). The whole extraction procedure was repeated twice and yielded 2 g of crude culture extract.

### 4.3. Isolation of Secondary Metabolites

The crude extract was purified by reversed-phase silica gel vacuum flash chromatography (Interchim, puriFlash^®^450, Montlucon CEDEX, France), using three consecutive Interchim puriFlash^®^ 32 g silica IR-50C18-F0025 flash columns (particle size: 50µm). The columns were eluted with a binary solvent gradient (solvent A: H_2_O, solvent B: ACN). The starting linear gradient from 10% B to 27% B during 25 min at a flow rate 15 mL/min was followed by an isocratic gradient at 52% B for 10 min. Then a linear gradient from 52% to 66% B over 7min was applied at the same flow rate and finally the column was washed starting with 100% B for 10 min followed by 100% A for 10 min at a flow rate 15–30 mL/min. UV 254 nm and UV scan 200–400 nm modes were used for detection and final separation of 5 main fractions (F1–F5), which were consequently concentrated under reduced pressure at 45 °C. The target compound was found in fraction F2 (16–18 R_t_, yield: 40 mg). It was resolved in a solvent mix (1:1:1; ACN/CH_3_OH/H_2_O) and further purified by an Agilent 1260 Infinity preparative HPLC (Agilent, Santa Clara, CA, USA) on a reversed phase column Gemini NX C-18 (21.20 × 150 mm, 5 µm, 110 Å). Gradient starting with 30 % ACN and 70 % H_2_O up to 90 % ACN in 10min (total time 34 min) and a flow rate of 25 mL/min. Four fractions (pF1-pF4, time slice each 1 min) were collected, of which pF4 contained the target. Yield of target-luteapyrone in pF4 (t_R_ 4.4 min) after one stage of prep HPLC was 4.39 mg. For purity check (Figure S8), an Agilent 1200 system was used with the same stationary phase (Gemini 5 µm NX-C18 110 Å, 150 × 2 mm) and gradient program at a flow rate 0.3 mL/min.

### 4.4. LC-MS and NMR

A diluted solution of the purified metabolite was measured with liquid chromatography-high resolution mass spectrometry. Chromatographic separation was carried out with a reverse phase C18 column (Gemini ^®^, NX-C18, 5 µm, 110 Å, 150 × 2 mm, Phenomenex, Torrance, CA, USA) in an UHPLC-system (Vanquish-Thermo Fisher Scientific, Bremen, Germany). 5 µL of sample solution were injected and gradient elution was carried out using H_2_O and ACN each containing 0.1% formic acid (FA) as eluent A and B respectively. The flow rate was set to 0.3 mL/min and the column was kept at 25 °C. After two minutes of linear elution with 15% B, a 30 min gradient to 95% B followed by three minutes constant 95% B and re-equilibration of the system with 15% B for ten minutes was applied resulting in a chromatographic method of 45 min. The UHPLC-system was coupled to a QExactive HF Orbitrap mass spectrometer (Thermo Fisher Scientific, Bremen, Germany) via a heated ESI interface operating in fast-polarity switching mode (positive/negative ionization). Full MS/TopN MS/MS scan events using an inclusion list were carried out for the positive and negative ionization mode. Full scan mass spectra were recorded in profile mode with a scan range *m/z* 100–1000 and a resolution of 120,000 FWHM (at *m/z* 200). If ions listed in the inclusion list were present in the full scan mass spectra, MS/MS was triggered with an isolation window of *m/z* ± 1 and stepped collision energy (25, 35, 45 eV) in the HCD collision cell. MS/MS fragment spectra were recorded with a resolution setting of 15,000 FWHM (at *m/z* 200). Manual data evaluation was carried out with Thermo Scientific™ Xcalibur™ software.

All NMR spectra were recorded on a Bruker Avance II 400 (Rheinstetten, Germany) (resonance frequencies 400.13 MHz for ^1^H and 100.63 MHz for ^13^C) equipped with a 5 mm N_2_-cooled cryo probe head (Prodigy) with z-gradients at room temperature with standard Bruker pulse programmes. The sample was dissolved in 0.6 mL of MeOD (99.8% D) and a few drops of DMSO-*d*_6_ (99.8% D). Chemical shifts are given in ppm, referenced to residual solvent signals (3.31 ppm for ^1^H, 49.0 ppm for ^13^C). ^1^H NMR data were collected with 32k complex data points and apodized with a Gaussian window function (lb = −0.3 Hz and gb = 0.3 Hz) prior to Fourier transformation. ^13^C spectrum with WALTZ16 ^1^H decoupling was acquired using 64k data points. Signal-to-noise enhancement was achieved by multiplication of the FID with an exponential window function (lb = 1 Hz). All two-dimensional experiments were performed with 1k × 256 data points, while the number of transients (2–16 scans) and the sweep widths were optimized individually. HSQC experiment was acquired using adiabatic pulse for inversion of ^13^C and GARP-sequence for broadband ^13^C-decoupling, optimized for ^1^*J*_(CH)_ = 145 Hz. For the NOESY spectrum a mixing time of 0.8 s was used.

### 4.5. Biological Assays

#### 4.5.1. Antimicrobial Activity

Minimal inhibitory concentrations (MIC) were quantified according to EUCAST guidelines (http://www.eucast.org, accessed on: 20 October 2020). The following microorganisms were used: bacteria: *Staphylococcus aureus* ATCC 6538 (Gram-positive), *Escherichia coli* ATCC 25922, *Klebsiella pneumoniae* ATCC 10031 and *Pseudomonas aeruginosa* ATCC 9027 (Gram-negative); fungi: *Candida albicans* ATCC 10231, and the filamentous fungi *Aspergillus fumigatus* (RL578), *Fusarium oxysporum* (RL108), *Fusarium solani* (RL 585). Concentration range of the compound tested for the evaluation of the antimicrobial activity was 5–985 µM.

#### 4.5.2. Cytotoxicity Test

The human embryonic kidney cell line HEK-293 cells (obtained from ATCC) as well as the two human cancer cell lines, the epidermal carcinoma-derived cell line KB-3-1 (generously donated by Dr. Shen, Bethesda, MD, USA) and the colon carcinoma cell line CaCo-2 cells (obtained from ATCC) were used in this study. Cells were cultivated in Dulbecco’s modified Eagle medium (DMEM, GibcoTM by Life Technologies, LifeTech Austria, Vienna, Austria), supplemented with 5% fetal bovine serum (FBS, GibcoTM by Life Technologies, LifeTech Austria) and 1% penicillin-streptomycin (Sigma-Aldrich, Vienna, Austria) at 37 °C with 5% CO_2_ in a humidified incubator. Cultures were periodically checked for *Mycoplasma* contamination.

Cells were seeded in 96 well plates at a density of 5 × 10^4^ cells/mL in 100 µL per well and allowed to attach for 24 h. Afterwards, cells were incubated with 100 µL of luteapyrone diluted in DMEM at concentrations ranging from 10 to 320 µM. As the compound is poorly soluble in water, stock solutions were prepared in water with 10% (*v/v*) dimethyl sulfoxide (DMSO) and stored at 4 °C. The proportion of viable cells was determined after 72 h exposure to luteapyrone by 3-(4,5-dimethylthiazol-2-yl)-2,5-diphenyltetrazolium bromide (MTT)-based vitality assay (EZ4U, Biomedica, Vienna, Austria). Briefly, 20 µL of the EZ4U assay solution was added to each well. After 2 h of incubation the absorbance was measured by a microplate reader, at 450 nm with 620 nm as reference to reduce unspecific background values. All experiments were performed three times in triplicates. Concentration range of the compound tested for the evaluation of the cytotoxic activity was 5–320 µM. 

## 5. Conclusions

The filamentous fungus *Metapochonia lutea* BiMM-F96/DF4 was cultivated on solid medium on ca 80 plates (2 L) containing modified yeast extract sucrose medium for 14 days at 25 °C in the dark. The crude ethyl acetate extract (2 g) was purified by reverse-phase silica gel flash chromatography, followed by preparative HPLC to isolate 4.39 mg of a new γ-pyrone derivative, named luteapyrone (1). Its chemical structure was elucidated by NMR and LC-MS. Luteapyrone (1) was evaluated for its cytotoxic effects against the Caco-2, KB-3-1, and HEK-293 cell lines, and antimicrobial activity against eight selected pathogenic Gram-positive/negative bacteria and human pathogenic fungi. The compound did not display any activity in these assays. Due to its structural similarity to other physiologically active actinopyrones, its potential should be further investigated as an insecticidal, antiparasitic or even antiobesity agent.

## Figures and Tables

**Table 1:** NMR chemical shifts for luteapyrone (**1**) recorded in CD_3_OD at 400 MHz.

	^1^H	^13^C
2	-	164.7
3	-	100.0
4	-	183.2
5	-	119.0
6	-	159.5
7	3.48 (d, 2H, *J* = 7.4)	30.7
8	5.44 (tq, 3H, *J* = 7.4, 1.3)	122.5
9	-	134.5
10	3.05 (br.s, 2H)	45.7
11	-	175.7
12	1.81 (s, 3H)	7.1
13	1.95 (s, 3H)	10.0
14	1.86 (br.s, 3H)	16.8
OMe	4.02 (s, 3H)	56.5

## Data Availability

The data presented in this study are available in supplementary material and/or from the author.

## Supplementary Materials

The following are available online, Figure S1: ^1^H NMR spectra of 1, Figure S2: ^1^H NMR spectra of 1 (expansion), Figure S3: 13C NMR spectra of 1, Figure S4: ^13^C NMR spectra of 1 (expansion), Figure S5: COSY spectrum of 1, Figure S6: Edited HSQC spectrum of 1, Figure S7: HMBC spectrum of 1, Figure S8: HPLC of 1, Figure S9: Flash chromatography of 1, Table S1: Cytotoxic activity of 1, Table S2: Antimicrobial activity of 1.

## Sample Availability

Samples of the compound are available from the authors.
