# Supplementary material for: Luteapyrone, a Novel ƴ-Pyrone Isolated from the Filamentous Fungus Metapochonia lutea
Source: Molecules. 2021 Oct 30;26(21):6589. doi: 10.3390/molecules26216589 (PMC8588484; doi:10.3390/molecules26216589)
Supplement: Supplementary file 1 [file molecules-26-06589-s001.zip › molecules-1394950-supplementary.pdf]

Supplementary Information

Luteapyrone, a novel γ-pyrone isolated from the filamentous fungus *Metapochonia lutea*

Roman Labuda[a,b*], Markus Bacher[c], Hannes Gratzl[b,d], Maria Doppler[b,d], Alexandra Parich[d], Mohammed Aufy[e], Rosa Lemmens-Gruber[e], Rainer Schuhmacher[b,d], Kathrin Rychli[a,b], Martin Wagner[a,b], Thomas Rosenau[c,g], Joseph Strauss[b,f], Christoph Schüller[b,f]

Figure. S1: ^{1}H NMR spectra of **1**

Figure. S2: ^{1}H NMR spectra of **1** (expansion)

Figure. S3: ^{13}C NMR spectra of **1**

Figure. S4: ^{13}C NMR spectra of **1** (expansion)

Figure. S5: COSY spectrum of **1**

Figure. S6: Edited HSQC spectrum of **1**

Figure. S7: HMBC spectrum of **1**

Figure. S8: HPLC of **1**

Figure. S9: Flash Chromatography of **1**

Table S1. Cytotoxic activity of **1**

Table S2. Antimicrobial activity of **1**

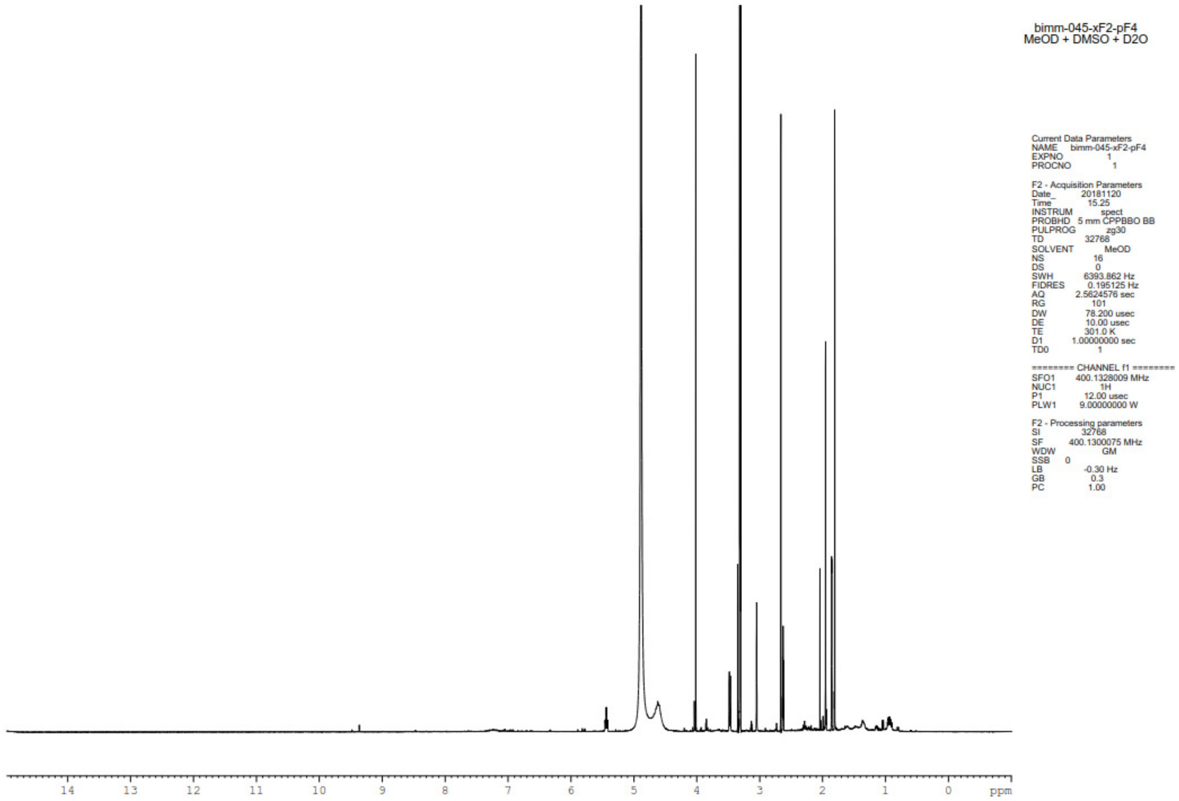

Figure. S1: ^{1}H NMR spectrum of **1**

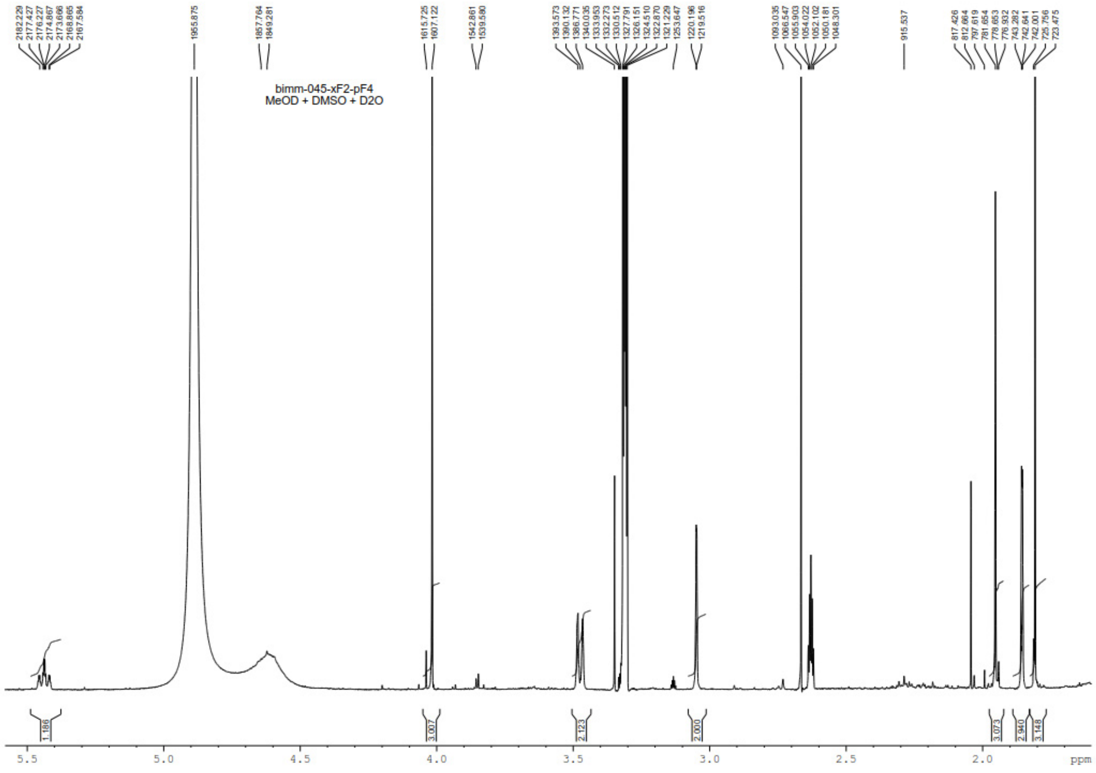

Figure. S2: ^{1}H NMR spectrum of **1** (expansion)

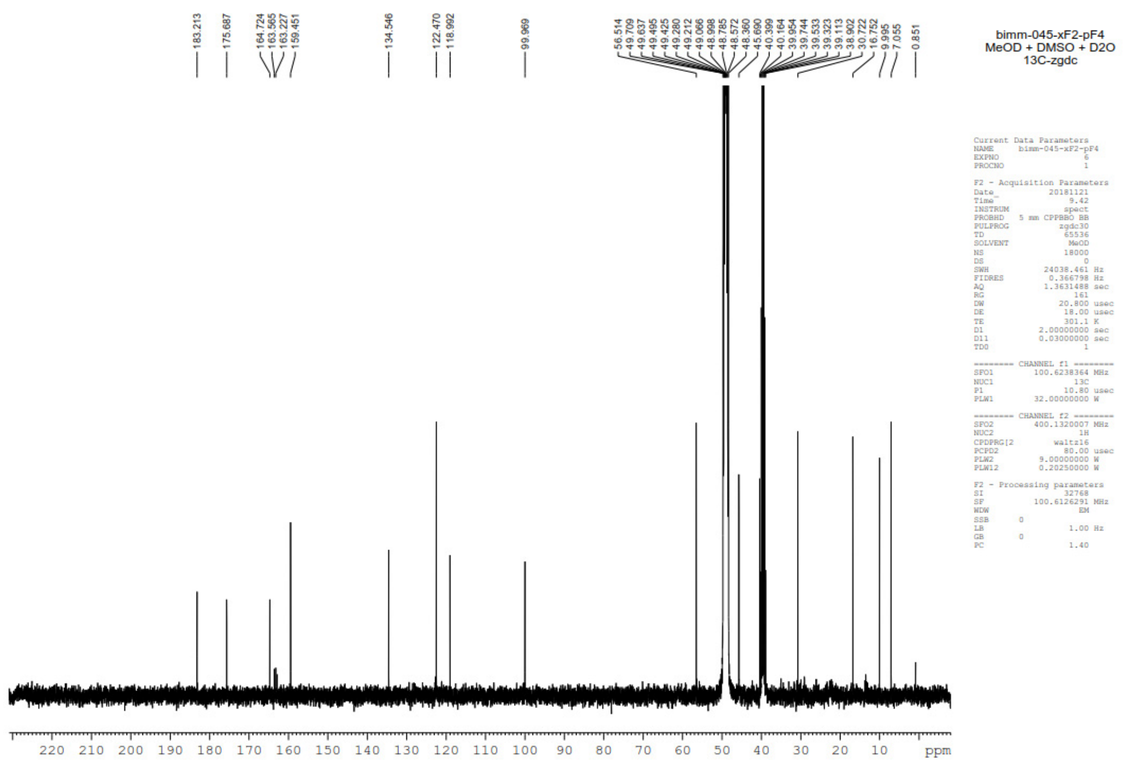

Figure. S3: ^{13}C NMR spectrum of **1**

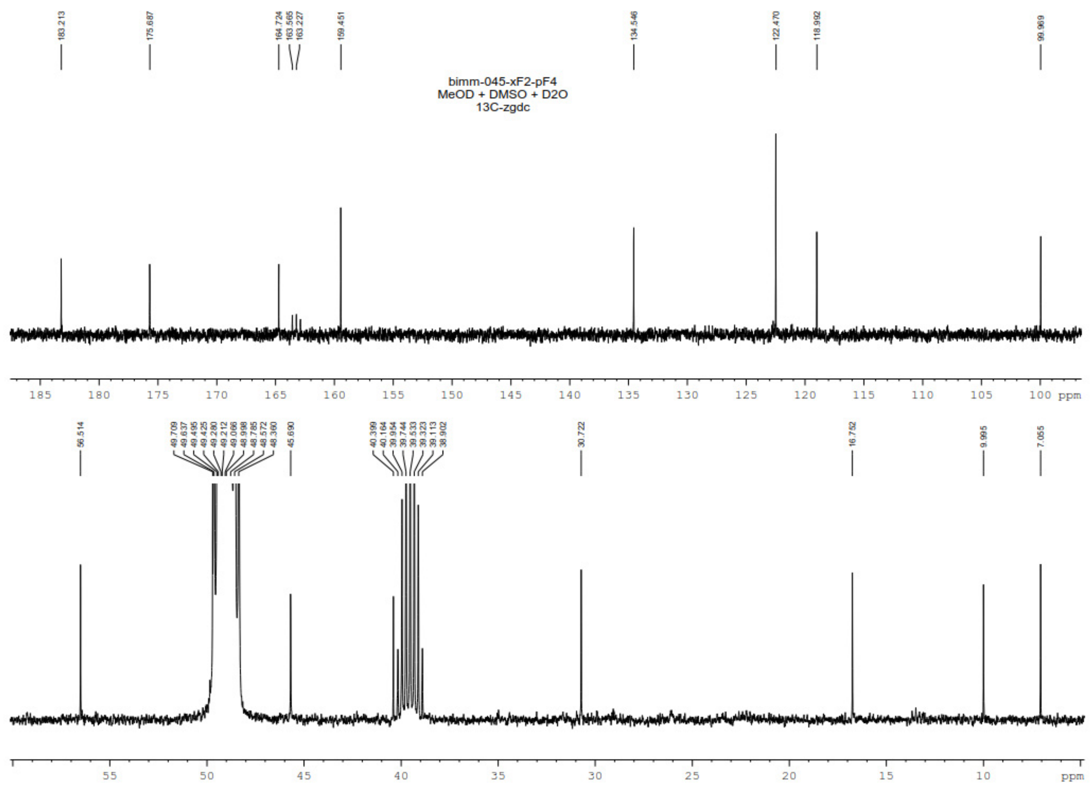

Figure. S4: ^{13}C NMR spectrum of **1** (expansion)

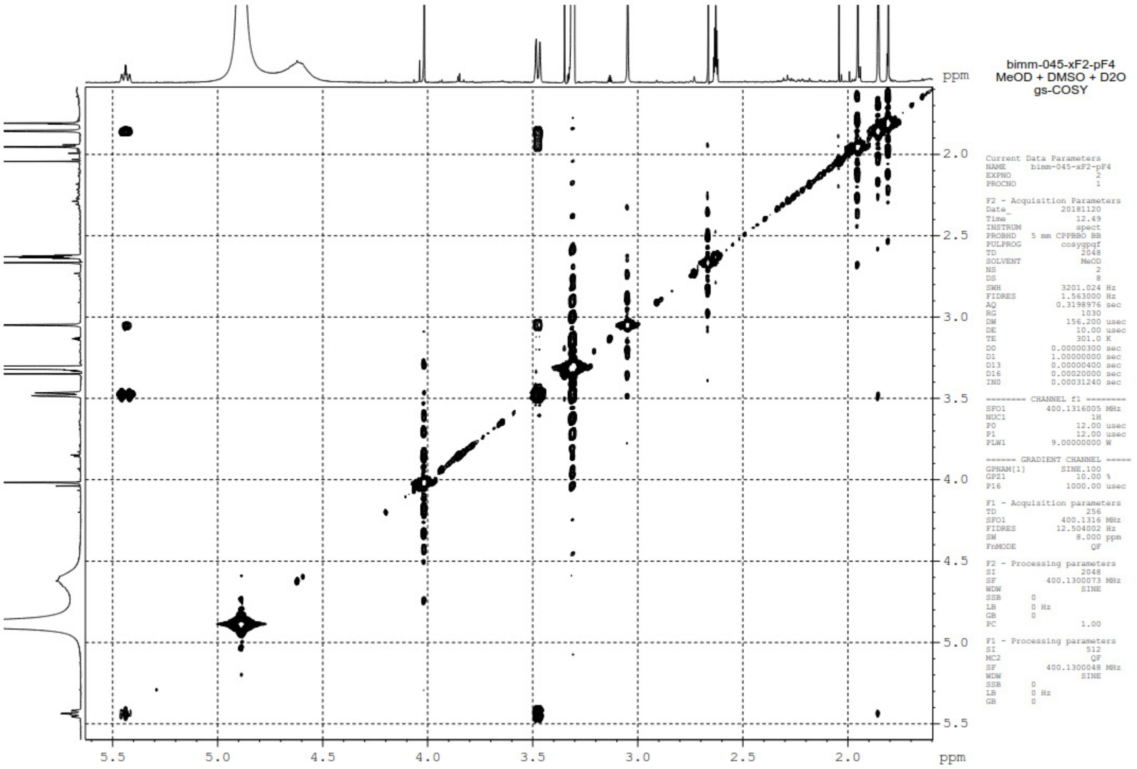

Figure. S5: COSY spectrum of **1**

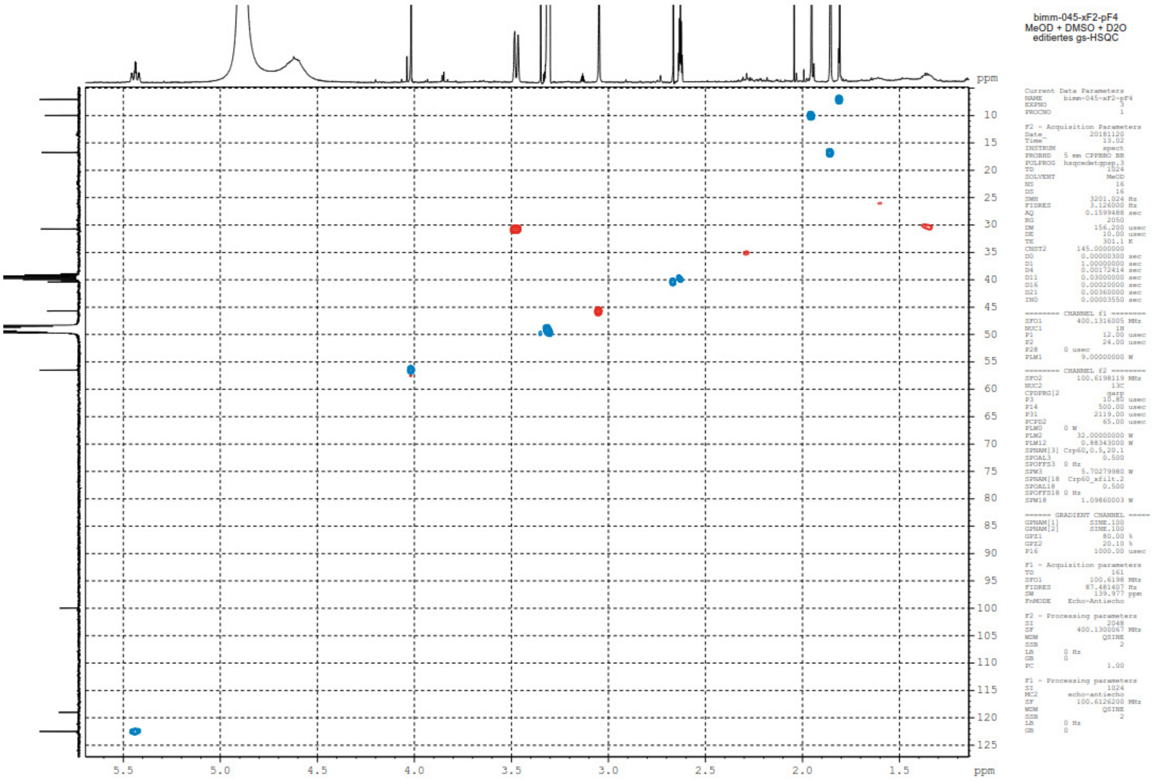

Figure. S6: Edited HSQC spectrum of **1**

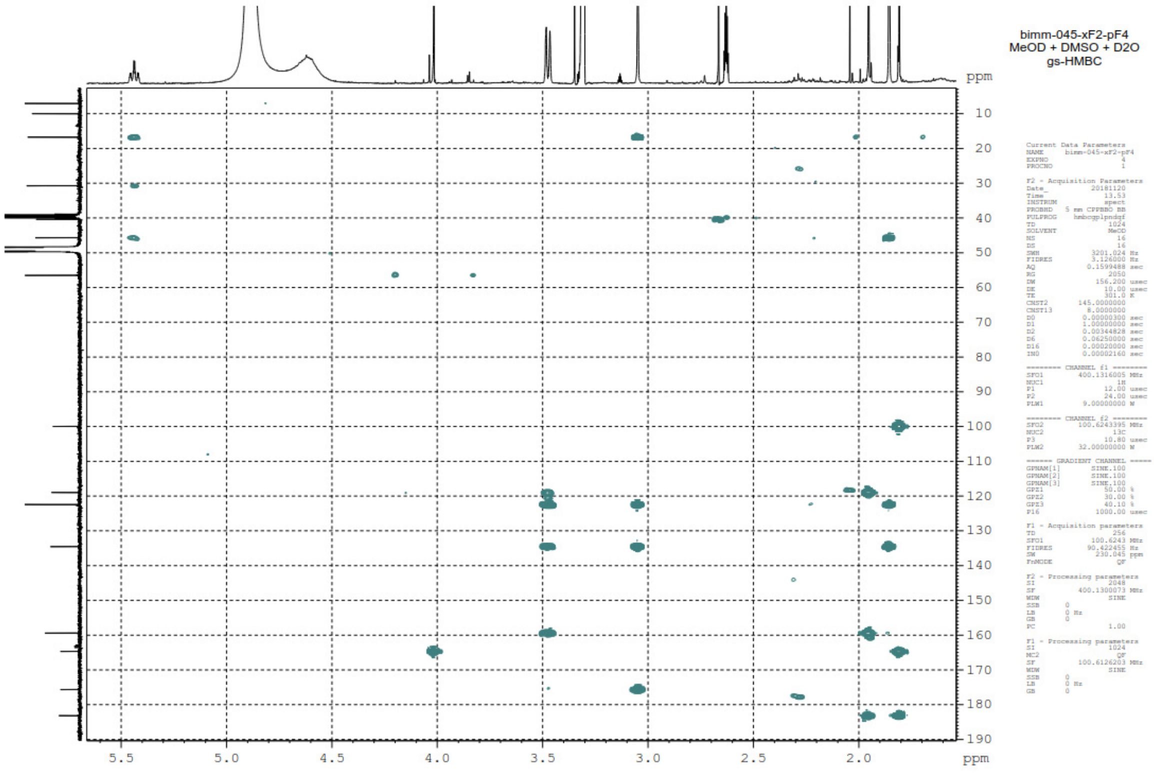

Figure. S7: HMBC spectrum of **1**

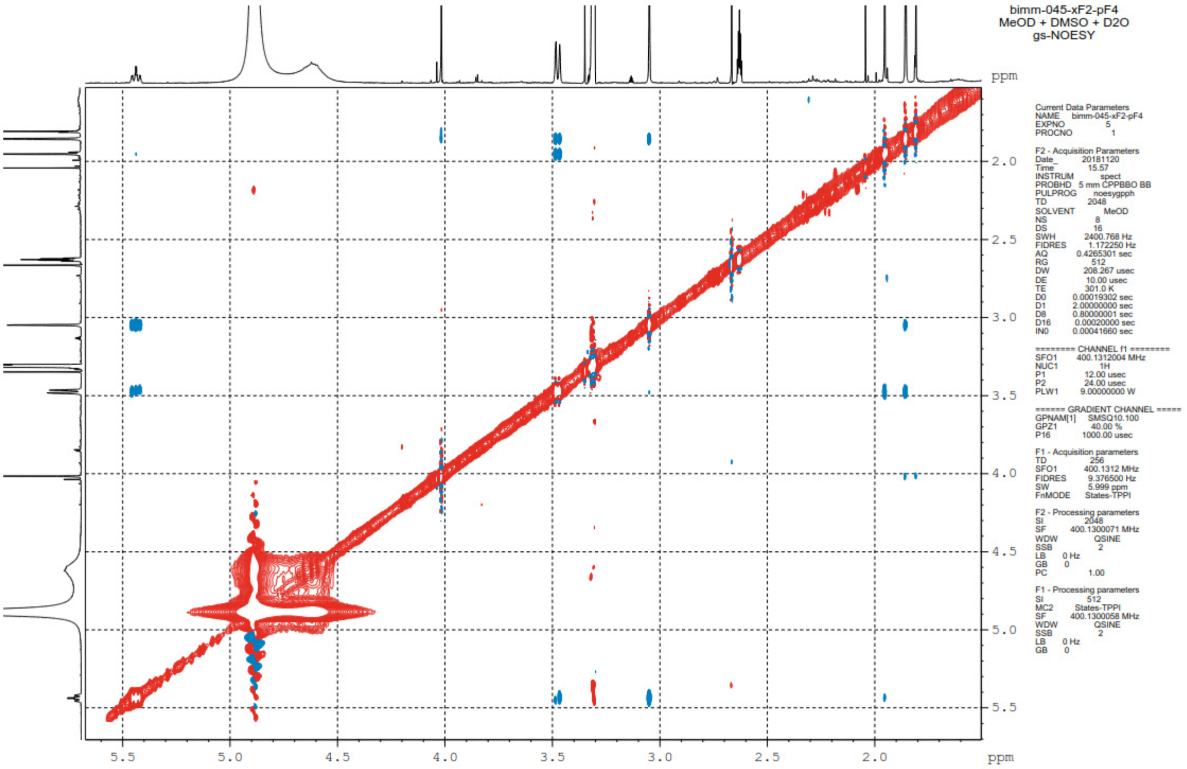

Figure. S8: HPLC of 1

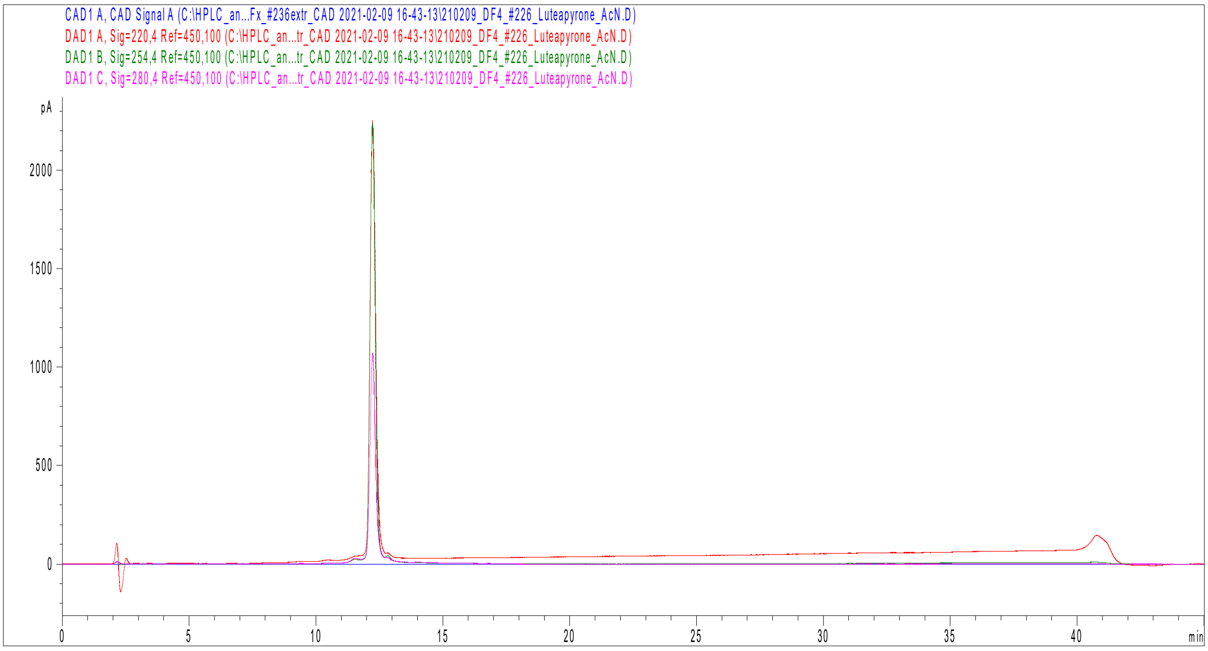

Figure. S9: Reveleris Flash Chromatography with Flow Rate Table

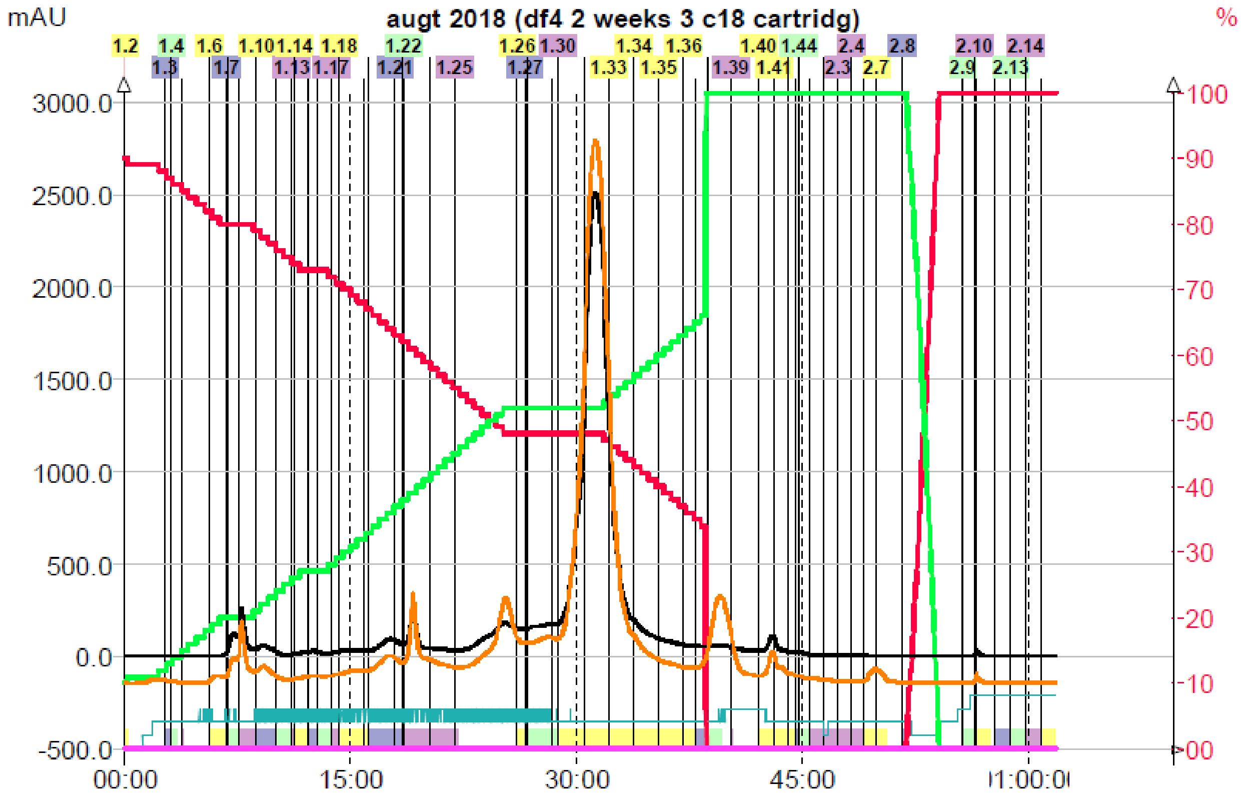

Fraction (F2) with Luteapyrone eluates at ca 16-18 min

Data Time : 9/28/2018 11:23:30 AM

180928-0945-augt 2018

Printing Time : 9/28/2018 12:26:56 PM

Elution Steps

N°	Time	Flow Rate	%A	%B
01	00 s	15.0	90	10
02	18 s	15.0	89	11
03	01:04	15.0	89	11
04	01:09	15.0	89	11
05	01:58	15.0	89	11
06	03:25	15.0	86	14
07	03:26	15.0	86	14
08	06:33	15.0	80	20
09	08:13	15.0	80	20
10	11:49	15.0	73	27
11	13:14	15.0	73	27
12	25:21	15.0	48	52
13	31:29	15.0	48	52
14	38:30	15.0	34	66
15	38:36	15.0	00	100
16	41:45	25.0	00	100
17	44:22	25.0	00	100
18	44:25	30.0	00	100
19	46:19	30.0	00	100
20	46:22	15.0	00	100
21	46:40	15.0	00	100
22	46:43	30.0	00	100
23	51:51	30.0	00	100
24	52:09	30.0	12	88
25	52:12	15.0	12	88
26	52:29	15.0	25	75
27	52:32	25.0	25	75
28	54:06	25.0	100	00
29	59:06	25.0	100	00
30	01:04:03	25.0	100	00
31	01:09:03	25.0	100	00

Detection Steps

N°	Time	Parameter	Collect	Threshold	F1
01	00 s	UV400:SIG1 ==> 254 nm	Yes	25	3
		UV400:SCAN ==> 200-400	Yes	5	3

Table S1. Cytotoxic activity of luteapyrone (5-320 μM)

**HEK-293**			**KB-3-1**			**Caco-2**		
Conc. (μM)	Control (DMSO)	Luteapyrone	Conc. (μM)	Control (DMSO)	Luteapyrone	Conc. (μM)	Control (DMSO)	Luteapyrone
5	2.879<br>2.341<br>2.226<br>***2.490***	2.351<br>2.631<br>2.761<br>***2.460***<br>(98.8%)	5	1.513<br>1.973<br>1.820<br>***1.769***	1.918<br>1.716<br>1.425<br>***1.686***<br>(95.3%)	5	2.635<br>2.611<br>2.352<br>***2.533***	2.660<br>2.228<br>2.413<br>***2.434***<br>(96.1%)
10	2.351<br>2.631<br>2.761<br>***2.581***	2.065<br>2.699<br>2.781<br>***2.515***<br>(97.4%)	10	1.660<br>1.685<br>1.609<br>***1.651***	1.532<br>1.781<br>1.627<br>***1.647***<br>(99.8%)	10	2.183<br>2.514<br>2.287<br>***2.328***	2.318<br>2.361<br>2.507<br>***2.395***<br>(102.9%)
20	2.348<br>2.565<br>2.310<br>***2.420***	2.618<br>2.660<br>2.327<br>***2.535***<br>(104.8%)	20	1.501<br>1.320<br>1.721<br>***1.514***	1.687<br>1.72<br>1.356<br>***1.590***<br>(105.0%)	20	2.720<br>2.719<br>2.524<br>***2.654***	2.087<br>2.245<br>2.249<br>***2.194***<br>(82.7%)
40	2.879<br>2.222<br>2.637<br>***2.579***	2.141<br>2.798<br>2.313<br>***2.417***<br>(93.7%)	40	1.280<br>1.660<br>1.536<br>***1.492***	1.500<br>1.570<br>1.498<br>***1.523***<br>(102.1%)	40	2.660<br>2.328<br>2.329<br>***2.439***	2.518<br>2.043<br>2.430<br>***2.330***<br>(95.5%)
80	2.470<br>2.388<br>2.308<br>***2.389***	2.678<br>2.416<br>2.415<br>***2.506***<br>(104.9%)	80	1.413<br>1.627<br>1.452<br>***1.497***	1.440<br>1.389<br>1.570<br>***1.466***<br>(97.9%)	80	2.321<br>2.128<br>2.587<br>***2.345***	2.230<br>2.814<br>2.042<br>***2.362***<br>(100.7%)
160	2.022<br>2.321<br>2.287<br>***2.210***	2.154<br>2.418<br>2.045<br>***2.196***<br>(99.4%)	160	1.256<br>1.213<br>1.061<br>**1.177**	1.236<br>1.330<br>1.111<br>**1.226**<br>(104.2%)	160	1.981<br>2.015<br>2.081<br>**2.026**	1.736<br>1.983<br>1.836<br>**1.852**<br>(91.5%)
320	2.063<br>1.987<br>2.105<br>***2.052***	2.218<br>2.067<br>2.157<br>***2.147***<br>(104.6%)	320	1.098<br>1.017<br>1.148<br>**1.088**	1.070<br>0.915<br>1.115<br>**1.033**<br>(94.9%)	320	1.698<br>1.618<br>1.826<br>**1.714**	1.552<br>1.483<br>1.313<br>**1.449**<br>(84.5%)

Table S2. Antimicrobial activity of luteapyrone

Microorganisms[a]	MIC (μM)
Staphylococcus aureus ATCC 6538	>985
Escherichia coli ATCC 25922	>985
Klebsiella pneumoniae ATCC 10031	>985
Pseudomonas aeruginosa ATCC 9027	>985
Candida albicans ATCC 10231	>985
Aspergillus fumigatus RL 578	>985
Fusarium oxysporum RL 108	>985
Fusarium solani RL 585	>985

[a]ATCC, American Type Culture Collection, Manasas, VA, USA; RL personal fungal collection of Roman Labuda
